# Fluid Biomarkers in Hereditary Spastic Paraplegia: A Narrative Review and Integrative Framework for Complex Neurodegenerative Mechanisms

**DOI:** 10.3390/genes16101189

**Published:** 2025-10-13

**Authors:** Lorenzo Cipriano, Nunzio Setola, Melissa Barghigiani, Filippo Maria Santorelli

**Affiliations:** 1 Molecular Medicine, IRCCS Fondazione Stella Maris, 56128 Pisa, Italy; lorenzocipriano1991@gmail.com (L.C.); nsetola94@gmail.com (N.S.); mely.b91@hotmail.com (M.B.); 2 Department of Molecular Medicine and Medical Biotechnology, University Federico II, 80131 Naples, Italy

**Keywords:** hereditary spastic paraplegia, fluid biomarkers, neurofilament light chain, brain-derived tau, remote monitoring, neurodegeneration

## Abstract

Background: Hereditary spastic paraplegias (HSPs) are a group of neurodegenerative disorders marked by progressive corticospinal tract dysfunction and wide phenotypic variability. Their genetic heterogeneity has so far limited the identification of biomarkers that are broadly applicable across different subtypes. Objective: We aim to define a balanced review on the use of biomarkers in HSP. Methods: This review focuses on fluid biomarkers already available in clinical or research settings—primarily validated in other neurodegenerative diseases—and assesses their potential translation to the HSP context. Biomarkers such as neurofilament light chain, brain-derived tau, glial fibrillary acidic protein, and soluble TREM2 reflect key converging mechanisms of neurodegeneration, including axonal damage, neuronal loss, and glial activation. These shared downstream pathways represent promising targets for disease monitoring in HSP, independently of the underlying genetic mutation. Results: An integrative framework of fluid biomarkers could assist in defining disease progression and stratify patients in both clinical and research settings. Moreover, recent advances in ultrasensitive assays and remote sampling technologies, such as dried blood spot collection, offer concrete opportunities for minimally invasive, longitudinal monitoring. When combined with harmonized multicenter protocols and digital infrastructure, these tools could support scalable and patient-centered models of care. Conclusions: The integration of already available biomarkers into the HSP field may accelerate clinical translation and offer a feasible strategy to overcome the challenges posed by genetic and clinical heterogeneity.

## 1. Introduction

Hereditary spastic paraplegias (HSPs) are a clinically and genetically heterogeneous group of inherited neurodegenerative disorders primarily characterized by progressive spasticity and weakness of the lower limbs due to a length-dependent degeneration of the corticospinal tracts [1]. The clinical presentation of HSPs varies widely and is traditionally classified into two major forms: the pure forms, which are confined to spastic paraparesis with or without mild urinary disturbances and vibration sense impairment, and the complex forms, in which spasticity is accompanied by additional neurological or systemic features such as ataxia, cognitive impairment, epilepsy, peripheral neuropathy, or optic atrophy [2].

Epidemiological studies estimate the prevalence of HSP between 2 and 6 per 100,000 individuals worldwide, although reported values vary widely (0.1–9.6 per 100,000) depending on study design, diagnostic criteria, and geographic region [3,4,5]. Recent registry-based data from England and Northern Ireland documented an increase from 2.83 per 100,000 in 2000 to 6.27 per 100,000 in 2021, suggesting that the frequency of diagnosed cases may be higher than previously assumed [3]. Beyond prevalence, the global burden of HSP lies predominantly in long-term disability rather than premature mortality. Although disease-specific disability-adjusted life year (DALY) estimates are not currently available in Global Burden of Disease datasets, the nature of HSP—with its slow but relentless progression, decades-long disease duration, and substantial motor impairment—indicates that the impact is driven mainly by years lived with disability (YLD). Patients often require continuous rehabilitative care, assistive devices, and long-term social support, which contributes to a disproportionately high healthcare and societal burden relative to the rarity of the condition [6].

HSPs exhibit diverse modes of inheritance, including autosomal dominant, autosomal recessive, and X-linked patterns. To date, over 90 genetic loci (designated SPG, for “spastic gait”) have been identified, reflecting the remarkable genetic complexity of these disorders [7]. Among the most prevalent forms are SPG4 (associated with variants in the *SPAST* gene, accounting for approximately 40% of autosomal dominant HSP cases), SPG3A (*ATL1* variants, ~10%), and SPG11 and SPG7, which are frequent among autosomal recessive forms [8].

Despite advances in genetic diagnostics, biomarkers for HSPs remain largely underdeveloped. Only in selected forms—such as SPG5, where elevated levels of oxysterols have been proposed—have potential disease-specific biomarkers been identified [9]. However, no biomarker to date has demonstrated sufficient sensitivity or specificity to serve as a universal indicator across the spectrum of HSPs.

In recent years, the search for biomarkers in neurodegenerative diseases has progressively shifted toward minimally invasive approaches. While cerebrospinal fluid (CSF) biomarkers have historically been central to diagnostic strategies, their invasiveness poses practical limitations. Consequently, increasing attention is being directed toward blood-based biomarkers, which offer a more accessible and less invasive alternative. Technological advancements now allow for the quantification of proteins at femtoliter concentrations in plasma, enabling the detection of subtle molecular changes associated with neurodegeneration. The aim of this review is to provide a focused synthesis of current knowledge on fluid biomarkers in HSP and to place this information within a broader neurodegenerative framework. While previous reviews have mainly summarized individual biomarkers or emphasized their role in more common disorders such as Alzheimer’s disease or amyotrophic lateral sclerosis, the present work specifically addresses their applicability to HSP, distinguishing between data directly available from HSP cohorts and findings extrapolated from other conditions. Building on this dual source of evidence, we suggest an integrative framework, grouping biomarkers according to their main downstream pathophysiological domains—axonal, neuronal, glial, or immune—and considering their complementary use rather than their isolated value. In doing so, we refer to a conceptual organization of biomarkers around shared final pathways of neurodegeneration, without implying unification of the diverse upstream genetic mechanisms underlying HSP. Within this perspective, particular attention is given to how pharmacokinetic properties and mechanistic relevance can guide the choice of biomarkers at different disease stages. The added value of this review lies not only in highlighting biomarkers individually, but also in integrating them into a coherent strategy that reflects the principal biological processes underlying HSP. To achieve this, the review is organized into sections that first outline the technological platforms available for biomarker detection, followed by a biomarker-by-biomarker analysis of their biological role, current evidence in HSP, and translational prospects. The discussion then integrates these insights into a critical appraisal of current limitations, future research priorities, and potential clinical applications.

## 2. Neuro-Biomarker Detection Across Fluids: A Technological Perspective

The quantification of neurological biomarkers has advanced significantly with the emergence of highly sensitive detection platforms capable of measuring central nervous system (CNS)-derived proteins in both CSF and peripheral blood. Historically, CSF is considered the optimal matrix for biomarker analysis due to its proximity to the CNS and reduced biological complexity. However, the development of next-generation analytical technologies has enabled the reliable detection of many biomarkers in plasma and serum (Table 1), expanding the feasibility of minimally invasive diagnostics and disease monitoring—an especially important shift for chronic, slowly progressive conditions such as HSP.

A wide range of platforms is now available for plasma biomarker detection, each with distinct advantages and limitations [10,11]. Traditional methods like ELISA and chemiluminescent immunoassays (CLIA) are widely used due to their simplicity and scalability, but they often lack the sensitivity required to detect low-abundance neurological proteins in blood [10,11,12]. Similarly, bead-based multiplexing platforms such as xMAP (Multi-Analyte Profiling, Luminex Corporation, Austin, TX, USA) have improved analytical throughput but suffer from reproducibility challenges across laboratories [11,12].

One of the most important advances in biomarker quantification has come with Single-Molecule Array (Simoa, Quanterix Corporation, Billerica, MA, USA) technology [13]. This digital immunoassay platform isolates individual antibody–antigen complexes in thousands of femtoliter-sized wells, where signal amplification occurs only when a target molecule is present. As a result, Simoa achieves femtogram-level sensitivity, making it uniquely suited to detect low-abundance biomarkers such as neurofilament light chain (NfL), phosphorylated tau (p-tau) isoforms, glial fibrillary acidic protein (GFAP), and inflammatory cytokines—all highly relevant to the pathophysiology of neurodegenerative diseases. Simoa also provides high specificity, low background noise, and good reproducibility, enabling its adoption in both research and clinical settings [14]. The U.S. FDA has recognized its impact by granting Breakthrough Device designation for the plasma p-tau181 Simoa assay in Alzheimer’s disease—an important milestone for blood-based diagnostics in neurology.

Complementary platforms also contribute meaningfully to biomarker research. Electrochemiluminescence (ECL)-based systems (e.g., MSD, Meso Scale Diagnostics, Rockville, MD, USA; Elecsys, Roche Diagnostics, Rotkreuz, Switzerland; Lumipulse G, Fujirebio Diagnostics, Tokyo, Japan) offer strong sensitivity and automation with broader accessibility in clinical labs. Meanwhile, mass spectrometry (MS)-based techniques such as LC-MS/MS and immunoprecipitation-MS (IP-MS) provide unmatched specificity and isoform discrimination, though they require complex workflows and infrastructure [10,11,12]. Together, these technologies are enabling a shift toward more sensitive, specific, and patient-centered approaches to biomarker-based monitoring in neurodegenerative diseases. In the case of HSP, their application may be especially valuable for capturing dynamic disease activity, detecting subclinical progression, and informing future therapeutic strategies.

**Table 1:** Comparative Summary of Technologies for Blood-Based Biomarker Detection.

Technology	Principles	Typical Limit of Detection (LoD)	Strengths	Weaknesses
Single-Molecule Array (Simoa)	Digital immunoassay that isolates individual antibody–antigen complexes in femtoliter wells for single-molecule detection.	~10–30 fg/mL (0.01–0.03 pg/mL) [13]	Ultra-high sensitivity (fg/mL); excellent specificity; low background noise; reproducibility; minimally invasive.	High equipment cost; limited clinical availability; complex instrument setup.
Electrochemiluminescence (ECL)	Combines electrochemical activation and luminescence for detecting antibody–antigen interactions.	~0.1–1 pg/mL (sub-pg/mL possible in optimized assays) [15]	High sensitivity; automation-compatible; suitable for multiplexing.	Requires specialized instruments; moderate cost; may need optimization for low-abundance proteins.
xMAP (Luminex)	Fluorescent microsphere-based multiplex platform analyzed via flow cytometry.	~0.05–0.1 pg/mL [16,17]	Multiplex capability; high throughput; good specificity.	Inter-lab variability; complex data analysis; high initial instrumentation cost.
LC-MS/MS	Chromatographic separation is followed by mass spectrometry based on mass-to-charge ratios.	~0.1–1 pg/mL (depending on enrichment and matrix) [18]	Unmatched specificity; distinguishes isoforms and PTMs; ideal for biomarker validation.	Technically demanding; expensive; low throughput; requires advanced lab infrastructure.
Immunoprecipitation + MS (IP-MS)	Enrichment of target proteins via antibodies followed by mass spectrometry analysis.	Typically pg/mL (single-digit pg/mL with enrichment) [19]	High specificity; allows discovery of interactomes and biomarker isoforms.	Labor-intensive; needs high-quality samples; complex protocols.
ELISA/CLIA	Antibody-based detection using enzyme-linked or chemiluminescent signal generation.	~1 pg/mL (commercial kits range 1–100 pg/mL) [20]	Widely available; automation possible; cost-effective for high-abundance targets.	Limited sensitivity in blood; narrow dynamic range; prone to interference from plasma matrix.

Abbreviations: Simoa: Single-Molecule Array; ECL: Electrochemiluminescence; xMAP: Multi-Analyte Profiling; LC-MS/MS: Liquid Chromatography–Tandem Mass Spectrometry; IP-MS: Immunoprecipitation–Mass Spectrometry; ELISA: Enzyme-Linked Immunosorbent Assay; CLIA: Chemiluminescent Immunoassay.

## 3. Targeting Molecular Pathways: A Biomarker-by-Biomarker Insight into HSP

Advances in fluid biomarker research have provided new tools to study and monitor complex neurodegenerative diseases such as HSP. While most available biomarkers were initially validated in conditions like Alzheimer’s disease (AD), amyotrophic lateral sclerosis (ALS), or frontotemporal dementia (FTD), growing evidence supports their broader applicability across disease spectra that share common pathological end points—such as axonal degeneration, neuronal injury, synaptic dysfunction, glial activation, and neuroinflammation [21,22]. In the case of HSP, a group of genetically and clinically heterogeneous disorders, this approach is particularly compelling, as no single biomarker is likely to capture its full biological complexity.

To structure this framework, we conducted a narrative review of the available literature, systematically searching for studies on fluid biomarkers in HSP and related neurodegenerative disorders. This article was conceived as a narrative review aimed at integrating current knowledge on fluid biomarkers in HSP with insights from related neurodegenerative conditions. Literature searches were conducted in PubMed up to July 2025. The following keywords and their combinations were used: “hereditary spastic paraplegia”, “paraplegia”, “paraparesis”, “spasticity”, “spastic”, “Strümpell–Lorrain”, “biomarkers”, “amyloid”, “tau”, “GFAP” (and “glial fibrillary acidic protein”), “sTREM2” (and “soluble TREM2”), “cytokines”, “interleukin”, “tumor necrosis factor”, “interferon”, “SNAP25” (and “synaptosomal-associated protein 25”), “PSD95” (and “postsynaptic density protein 95”), “TDP-43” (and “TAR DNA-binding protein 43”), “UCHL1” (and “ubiquitin carboxyl-terminal hydrolase L1”), as well as the names of the main HSP genes and SPG subtypes. We included peer-reviewed articles in English that reported original data on fluid biomarkers in HSP or, in the absence of HSP-specific studies, on related neurodegenerative disorders such as ALS, FTD, or AD. Reviews, case reports, and studies with very small cohorts (*n* < 5) were excluded unless they provided unique information relevant to HSP. Given the rarity of HSP and the limited availability of biomarker data, evidence from other diseases was considered when mechanistically relevant or methodologically informative

The following sections provide a systematic evaluation of commercially available biomarkers, focusing on their biological characteristics, detection platforms, and potential utility within the clinical and research context of HSP (Table 2). Biomarkers were termed as “established”, “preliminary”, or “speculative” according to the strength of evidence in HSP. “Established” is a term referred to case–control studies with concordant results; “preliminary” relates to single studies or inconsistent findings; “speculative” is used for markers proposed only on mechanistic grounds (mostly derived from other neurodegenerative conditions) without direct evidence in HSP. Each is examined in relation to its biological role, detection modality, and translational potential, with attention to how these markers may reflect different facets of HSP pathology—ranging from chronic axonopathy to transient inflammatory or glial responses. This includes well-established markers such as NfL, tau, and GFAP, as well as emerging candidates like BD-tau, sTREM2, UCHL1, and synaptic proteins including PSD-95 and SNAP-25. Inflammatory cytokines are also discussed as potential modulators or indicators of disease activity. Collectively, these biomarkers form the foundation for a multidimensional framework that could inform both clinical care and research, guiding early diagnosis, tracking disease progression, and enabling tailored therapeutic strategies in HSP.

### 3.1. Amyloid (Aβ40 and Aβ42)

Amyloid-β (Aβ) peptides, particularly Aβ42, are central to the pathophysiology of AD and are widely used as core biomarkers in that context [45,46,47]. By contrast, alterations in amyloid processing are not part of the classical mechanisms underlying HSP, and significant changes in Aβ42 or Aβ40 are not expected. This has been confirmed upon testing patients with the SPG4 form. Indeed, there were no notable differences in CSF levels of Aβ peptides in a recent study of a preSPG4 cohort of patients [48]. Rare exceptions exist in HSP phenotypes associated with variants in genes directly involved in amyloid processing (e.g., *PSEN1*), where abnormal Aβ42/Aβ40 ratios have been reported [49,50]. However, these represent isolated overlaps with AD pathology and are not relevant for biomarker discovery aimed at tools broadly applicable across the HSP spectrum. For this reason, amyloid biomarkers are not recommended for diagnostic or monitoring purposes in HSP.

### 3.2. Tau (Total, Brain-Derived and Phosphorylated)

Tau is a microtubule-associated protein essential for axonal stability and transport [51,52]. In AD, phosphorylated tau isoforms such as pTau181, pTau217, and pTau231 are widely used as biomarkers [53,54], but the amyloid-driven cascade that leads to tau pathology is not relevant to HSP [55,56]. Accordingly, phosphorylated tau forms are unlikely to have utility in this context. Total tau (t-tau) and especially brain-derived tau (BD-tau), which selectively detects CNS-originating tau, may serve as exploratory markers of neuronal injury in advanced or complex HSP subtypes [26,57], although evidence is still very limited [48,51,52,53,54,55,56,57]. In the preSPG4 cohort [48], no significant differences in CSF levels of t-tau or phosphorylated tau were observed between SPG4/*SPAST* mutation carriers and controls [48], consistent with the view that tau alterations are not a feature of the prodromal phase of SPG4. Whilst negative findings may also reflect methodological factors, including the small sample size and cross-sectional design, CNS-specific forms like BD-tau remain largely unexplored in HSP. BD-tau could theoretically offer an advantage over conventional plasma t-tau in capturing CNS-specific neurodegeneration in complex HSP phenotypes or in detecting disease conversion, but this requires validation, and multicenter longitudinal studies.

### 3.3. Neurofilament Light Chain

Neurofilament light chain (NfL) is a structural cytoskeletal protein predominantly expressed in large-caliber myelinated axons, where it contributes to axonal stability, diameter, and conduction. Upon axonal injury or degeneration, NfL is released into the interstitial fluid and subsequently measurable in CSF and blood, making it a sensitive marker of neuroaxonal damage. Clinically, NfL has been established as a key biomarker for disease activity, progression, and treatment response in conditions such as multiple sclerosis, amyotrophic lateral sclerosis, frontotemporal dementia, and AD [58]. It is currently used in multiple sclerosis to monitor disease activity and treatment response, in amyotrophic lateral sclerosis for prognosis and progression tracking, and in AD and FTD to support differential diagnosis and predict cognitive decline. Its levels also respond to acute insults such as traumatic brain injury or stroke and are increasingly included in the design and evaluation of clinical trials, serving as a pharmacodynamic marker.

In HSP, NfL has shown promise as a fluid biomarker of disease activity and progression. Wilke et al. first demonstrated elevated serum NfL levels in patients with genetically confirmed HSP compared to healthy controls [59]. Kessler et al. further characterized NfL dynamics in SPG4, showing modest elevations that reflected ongoing axonal degeneration but lacked strong correlation with clinical severity [34,60]. Importantly, the preSPG4 study by Rattay et al. found that while total tau and phospho-tau did not differentiate between mutation carriers and non-carriers, sNfL levels were significantly increased in symptomatic carriers compared to non-carriers and controls [48]. Notably, presymptomatic carriers had sNfL levels comparable to controls, suggesting that NfL elevation aligns with the clinical transition to symptomatic stages. In a recent pilot study on children with IAHSP, pNfL levels were found to be slightly but significantly elevated compared to age-matched healthy controls [61]. These findings support the presence of ongoing axonal degeneration in ALS2-related disorders. Notably, increased NfL levels were observed across a range of clinical severity, from milder to more advanced motor impairment, suggesting that NfL may serve as a useful biomarker for disease activity and progression in IAHSP. Despite the limited sample size, the consistency of the data highlights the potential role of NfL as a fluid biomarker for these ultra-rare neurodegenerative conditions.

Among fluid biomarkers, NfL are by far the most extensively studied in HSP, and they represent the markers for which the largest body of evidence is currently available. Early work (e.g., Wilke et al.) reported higher serum NfL in genetically confirmed HSP versus controls, supporting sensitivity to axonal injury, but the small, cross-sectional cohort and potential ascertainment biases limit generalizability [59]. In SPG4, Kessler et al. observed only modest elevations with weak correlations to clinical measures; inference is constrained by the single-genotype focus, small sample size, wide dispersion of ages and disease durations, and absence of longitudinal trajectories [34,60]. The preSPG4 study showed that sNfL is increased in symptomatic carriers but not in presymptomatic carriers, consistent with rises around clinical conversion; however, confirmation in larger and longitudinal datasets is still required [48]. Slight but significant elevations have also been described in ultra-rare pediatric forms (e.g., HPDL-SPG83- or ALS2-related disorders), again in very small cohorts. Across studies, pre-analytical factors (serum vs. plasma, platform differences) and, critically, the strong age-dependence of NfL complicate interpretation: NfL increases with healthy aging, so analyses should incorporate age-adjusted norms and explicit statistical correction for age (and ideally disease duration) or use age-normalized z-scores and within-subject longitudinal change to reduce confounding.

These findings suggest that sNfL may serve as a non-invasive biomarker to monitor disease progression, detect phenoconversion, and support stratification in future interventional trials. While its current limitations include lack of specificity, overlap with normal aging, and low sensitivity in early-stage or slowly progressive cases, its accessibility, reproducibility, and sensitivity to axonal damage render it a valuable tool in the clinical management and research of HSP.

### 3.4. Glial Fibrillary Acidic Protein

GFAP is a type III intermediate filament protein selectively expressed by astrocytes [62]. It plays a critical role in maintaining cytoskeletal integrity, responding to injury, and regulating blood–brain barrier development and astrocyte-neuron communication [63]. GFAP is a well-established marker of astrocyte activation and reactive gliosis. In neurodegenerative and neuroinflammatory disorders, elevated serum or CSF GFAP indicates glial stress or damage [64].

Astrocytes are central players in the maintenance of CNS homeostasis, supporting neuronal function through metabolic regulation, synaptic modulation, and preservation of blood–brain barrier (BBB) integrity. In neurodegenerative diseases, astrocytes undergo profound phenotypic changes, becoming “reactive” in response to injury, inflammation, or neuronal dysfunction [65]. This process, termed reactive astrogliosis, is characterized by hypertrophy, altered gene expression, and the secretion of both neurotoxic and neuroprotective molecules. Sustained astrocyte reactivity contributes to chronic neuroinflammation, synaptic dysfunction, and neuronal loss in a range of neurodegenerative disorders, including AD and ALS [66,67]. Astrocytes also play a critical role in maintaining BBB integrity through their endfeet interactions with endothelial cells. Disruption of this astrocyte-endothelium crosstalk can lead to increased BBB permeability, allowing infiltration of peripheral immune cells and reactive oxygen species that further exacerbate neurodegeneration. These mechanisms position astrocytic dysfunction as both a consequence and a driver of CNS pathology.

In the context of HSP, astrocytes are emerging as active contributors to disease pathogenesis [68]. In SPG3A, caused by pathological variants in the *ATL1* gene, astrocytes derived from patient iPSCs show defective lipid metabolism, including reduced cholesterol efflux and smaller lipid droplets [69]. This metabolic impairment leads to non–cell-autonomous axonal degeneration of cortical projection neurons—an effect that can be rescued by enhancing cholesterol transfer with LXR agonists. These findings reveal a direct pathological interaction between astrocytes and neurons in HSP, previously overlooked.

Beyond SPG3A, evidence is accumulating that lipid dysregulation and astrocytic dysfunction may also play a role in other HSP subtypes, such as SPG4, SPG11, and SPG31, where alterations in cholesterol handling, vesicle trafficking, and glial signaling have been reported [68]. Although the exact contribution of reactive astrogliosis to HSP remains incompletely understood, these data highlight astrocytes as potential mediators of neurodegeneration and support the use of astrocyte-derived biomarkers—such as GFAP—to quantify glial activation and monitor disease activity in selected HSP phenotypes.

Preliminary evidence on GFAP as a biomarker in HSP and related disorders has produced mixed findings, largely influenced by cohort size, genotype, and disease complexity. In a recent study, Kessler et al. compared serum levels of GFAP and NfL among individuals with primary progressive multiple sclerosis (PPMS), HSP, and healthy controls. While pNfL was mildly elevated in HSP, GFAP levels did not significantly differ between the HSP group and controls. However, interpretation is limited by the small sample size and the exclusive inclusion of SPG4 cases—a subtype typically associated with a pure motor phenotype and minimal glial activation [34] and dispersion of values across patients. This distribution suggests that factors such as age, disease duration, and interindividual variability may have masked more subtle differences, highlighting the need for larger and more homogeneous cohorts.

In contrast, a study of children with biallelic variants in the *HPDL* gene—associated with early-onset, complex motor disorders—revealed significantly elevated plasma GFAP levels compared to age- and sex-matched controls [70]. GFAP concentrations were notably higher in individuals with more severe neurological phenotypes, and a trend toward correlation with clinical severity was observed. These findings suggested that GFAP might reflect astrocytic activation and disease burden in pediatric-onset disorders with broader neurodegenerative involvement, supporting its potential role as a biomarker in selected, more complex HSP-related conditions. Taken together, current evidence points to a potential role for GFAP as a biomarker of glial activation in selected subtypes, while its overall utility in HSP will require longitudinal studies in larger, genotype-diverse, cohorts that account for age-related changes and other confounding factors.

### 3.5. Soluble Triggering Receptor Expressed on Myeloid Cells 2

sTREM2, the soluble form of Triggering Receptor Expressed on Myeloid cells 2, is released from the membrane of activated microglia via proteolytic cleavage [71]. It functions as a key regulator of microglial activation, survival, and phagocytosis, and plays a critical role in the innate immune response within the CNS [37]. Increased CSF sTREM2 has been observed in several neurodegenerative conditions, including AD, where it correlates with tau pathology and disease progression [72].

Microglia, the innate immune sentinels of the CNS, have emerged as central mediators of neuroinflammation and neurodegeneration in a wide range of neurological disorders [73]. Under physiological conditions, microglia survey the brain microenvironment, maintains synaptic architecture, and clear apoptotic debris [74]. In response to injury, protein aggregates, or metabolic stress, microglia undergo activation characterized by morphological remodeling, upregulation of inflammatory genes, and the release of cytokines, chemokines, and reactive oxygen species [73]. While this response can be protective in acute contexts, chronic microglial activation often contributes to synaptic loss, axonal degeneration, and neuronal dysfunction, making it a key driver of disease progression in neurodegenerative settings.

In HSP, the role of microglia has only recently begun to be defined. Compelling evidence from studies in SPG11, one of the most common and severe complex HSP subtypes, suggests that microglial activation is not a mere bystander effect but a direct contributor to disease pathogenesis. Postmortem analyses and iPSC-derived microglia from SPG11 patients show extensive microgliosis, pro-inflammatory gene signatures, and aberrant interferon gamma (IFNγ)-STAT1 signaling [75]. Moreover, animal models of SPG11 (e.g., *Spg11*^−/−^ mice) display both CNS-restricted microglial activation and peripheral immune dysregulation, including increased CD8^+^ T cell infiltration, suggesting a dysfunctional immune–glial axis [76]. More recently, similar immune-glial mechanisms have been observed in SPG15, another complex form of HSP. In *Zfyve26*^−/−^ mice, fate-mapping and single-cell profiling revealed the early expansion of disease-associated microglia along with infiltration and local proliferation of CD8^+^ effector T cells, even prior to detectable neuronal loss [77]. Bidirectional signaling between microglia and T cells was suggested by cell–cell interaction analyses, indicating that crosstalk between these compartments may actively drive disease progression. These findings reinforce the concept that neuroinflammation in HSP is not simply reactive but may precede and promote neurodegeneration through maladaptive immune interactions.

Given the emerging evidence of microglial involvement in complex forms of HSP, microglia-derived biomarkers such as soluble TREM2—a cleavage product of the TREM2 receptor—may represent a potential exploratory tool for future studies. sTREM2 has demonstrated utility in other neurodegenerative diseases as a marker of microglial activation and phagocytic function, and although not yet studied in HSP, it represents a promising candidate for capturing neuroinflammation in subtypes like SPG11 and SPG15, detecting subclinical disease activity in presymptomatic carriers, and stratifying patients based on immune-glial engagement. When integrated with markers of axonal (NfL), neuronal (BD-tau), or astrocytic (GFAP) damage, sTREM2 could enhance multimodal biomarker strategies aimed at tracking disease progression and therapeutic response. No studies have yet investigated sTREM2 in HSP, and therefore its role remains entirely speculative.

### 3.6. Ubiquitin Carboxy-Terminal Hydrolase L1

Ubiquitin carboxy-terminal hydrolase L1 (UCHL1) is a neuron-specific deubiquitinating enzyme that plays a crucial role in maintaining proteostasis by regulating the ubiquitin-proteasome system. It is highly expressed in the cytoplasm of neurons, especially within axons and presynaptic terminals, making it a marker of neuronal and axonal integrity [78]. Upon neuronal injury, UCHL1 is released into the extracellular space and becomes detectable in CSF and, to a lesser extent, in blood, where it serves as a sensitive indicator of CNS injury.

UCHL1 has been validated as a biomarker of acute neuronal damage, particularly in traumatic brain injury (TBI). It is one of the two FDA-approved biomarkers (alongside GFAP) included in the Banyan Brain Trauma Indicator test for the early assessment of mild TBI [79]. Elevated UCHL1 levels in CSF and serum have also been reported in subarachnoid hemorrhage, spinal cord injury, and other acute CNS pathologies [78]. In these contexts, UCHL1 reflects cytoskeletal disruption and neuronal cell body damage, with concentrations correlating with injury severity and clinical outcome. To date, there are no published studies directly assessing UCHL1 in HSP, and it was not included in the biomarker panels evaluated in the preSPG4 cohort or related natural history studies [48]. This may reflect the slow progression of axonal degeneration in HSP, in contrast to the acute neuronal injury paradigms in which UCHL1 has been most useful. Nonetheless, given its high neuronal specificity and rapid release following neuroaxonal damage, UCHL1 could hold potential in complex or aggressive HSP variants, particularly where additional neurodegenerative mechanisms or gray matter involvement is suspected.

From a clinical perspective, UCHL1 might be considered for exploratory assessment in HSP as a complementary biomarker in HSP to detect or confirm active neurodegeneration, stratify patients based on disease severity, or monitor therapeutic interventions. However, its low sensitivity in chronic, progressive disorders and limited penetration into blood from CNS sources remain major challenges. At present there are no data supporting the use of UCHL1 in HSP, and its utility is likely limited by the chronic and slowly progressive nature of the disease. While neuronal specificity makes it a potential candidate, especially in rapidly progressive complex variants, its role in HSP still remains speculative.

### 3.7. TAR DNA-Binding Protein 43

TDP-43 (TAR DNA-binding protein 43) is a ubiquitously expressed nuclear protein involved in RNA metabolism, including splicing, transport, stability, and stress granule dynamics. In healthy neurons, it is predominantly localized in the nucleus, but under pathological conditions it mislocalizes to the cytoplasm, where it forms insoluble aggregates that are hyperphosphorylated, ubiquitinated, and cleaved. This mislocalization and aggregation process is a pathological hallmark of several neurodegenerative diseases, most notably amyotrophic lateral sclerosis and frontotemporal dementia [80,81]. TDP-43 pathology defines a class of diseases known as TDP-43 proteinopathies, in which its nuclear depletion and cytoplasmic accumulation contribute directly to neurodegeneration. In ALS and FTD, TDP-43 pathology is observed in the vast majority of cases, making it a central pathological feature [39,40]. However, detecting pathological TDP-43 in vivo remains challenging. Attempts to measure total or phosphorylated TDP-43 in biofluids such as CSF or plasma are ongoing, but current assays do not yet offer sufficient specificity to distinguish pathological from physiological forms [40]. As a result, TDP-43 is not currently used as a fluid biomarker in clinical settings.

In the context of HSP, data on TDP-43 are scarce but suggest intriguing connections. A well-documented case of SPG6, caused by a variant in the *NIPA1* gene, revealed widespread TDP-43 pathology at autopsy, including characteristic cytoplasmic inclusions in spinal motor neurons and cortical regions [82]. The patient had a complex HSP phenotype overlapping with motor neuron disease. While this remains an isolated finding, it raises the possibility that in some HSP subtypes—particularly those with atypical or multisystem involvement—TDP-43 may contribute to disease mechanisms. However, broader analyses have not identified *NIPA1* pathogenic variants as common in ALS, suggesting that the overlap between TDP-43 pathology and HSP is not generalized but may be restricted to specific genetic or phenotypic contexts.

To date, TDP-43 has not been investigated as a biomarker in multiple HSP cohorts, and it was not included in the biomarker panels analyzed in recent longitudinal studies [48]. Nonetheless, TDP-43 could, in principle, be of potential relevance in complex or overlapping phenotypes (e.g., HSP with cognitive impairment or lower motor neuron signs), although this remains unproven and requires confirmation in dedicated studies. Advances in assay development—particularly those targeting phosphorylated or truncated forms of TDP-43—may eventually enable its inclusion in multimodal biomarker strategies aimed at refining diagnosis, tracking progression, or stratifying phenotypes within the heterogeneous spectrum of HSP [81]. At present, evidence for TDP-43 in HSP remains hypothetical since the analogies with ALS/FTD and by anecdotal findings in SPG6/*NIPA1*.

### 3.8. Synaptic Biomarkers in HSP: Potential Roles and Preliminary Evidence

Although axonal degeneration remains the hallmark of HSP, increasing evidence suggests a possible contribution of synaptic dysfunction in certain complex forms. This hypothesis stems from neuropathological and experimental data implicating impairments in vesicle trafficking, cytoskeletal architecture, and membrane dynamic processes crucial to synaptic maintenance [55]. Synaptic biomarkers such as PSD-95 (a postsynaptic scaffolding protein) and SNAP-25 (a presynaptic SNARE component) have been proposed as indicators of synaptic integrity in other neurodegenerative diseases and may offer translational potential in HSP. Recent studies on SPG15 have shown that loss of spastizin disrupts synaptic structure and renders neurons more susceptible to excitotoxic stress [83]. Similarly, in SPG11, dysfunction of spatacsin results in abnormal synaptic vesicle trafficking, impaired axonal transport, and reduced expression of motor and synaptic genes, including SNAP-25 [84]. PSD-95 levels, however, appeared preserved in SPG11 iPSC-derived neurons, suggesting selective vulnerability within synaptic components. While these findings point to synaptopathy as a plausible secondary mechanism in HSP pathogenesis, further validation is required. Biomarkers such as PSD-95 and SNAP-25—particularly when measured in cerebrospinal fluid using ultrasensitive assays—may help detect synaptic involvement in vivo, as it is plausible from experimental work in SPG11 and SPG15. Future testing of synaptic biomarkers in biofluids will need rigorous studies in broader HSP genotypes essential to determine specificity, sensitivity, and clinical utility.

### 3.9. Cytokines

Cytokines are key mediators of inflammation and play a crucial role in the pathophysiology of neurodegenerative diseases. Interleukin-5 (IL-5), a Th2-associated cytokine, has recently been highlighted for its potential neuroprotective role in conditions such as ALS [85]. Higher IL-5 levels have been associated with slower disease progression and more favorable clinical patterns, suggesting that IL-5 may help modulate neuroinflammation by promoting an anti-inflammatory environment. In contrast, interferon-alpha (IFN-α), a type I interferon, is often linked to detrimental effects in neurodegenerative disorders [86]. It activates the JAK-STAT signaling pathway, leading to the expression of proinflammatory genes and contributing to neuronal dysfunction and degeneration.

Recent evidence in SPG11 supports a role for immune dysregulation and chronic neuroinflammation [76]. In particular, SPG11 patients exhibit increased peripheral levels of proinflammatory cytokines, including IL-6, TNF-α, and IFN-γ, as well as activation of IFN-γ–STAT1 signaling in iPSC-derived microglia and postmortem CNS tissue. These findings indicate that systemic and CNS-compartmentalized inflammation are active contributors to disease pathogenesis in SPG11, potentially mediated through sustained microglial activation and maladaptive T cell responses.

Furthermore, cytokines may, in theory, capture transient or subclinical inflammatory events that escape clinical detection but influencing the overall disease trajectory. It has been hypothesized that such episodic immune activations might contribute to subtle alterations in neuronal function or axonal integrity with immune–glial dysregulation being implicated in disease mechanisms at certain stages, at least in SPG11 disease [76]. These alterations could accumulate over time and shape the chronic degenerative process typical of HSP. Therefore, monitoring cytokine fluctuations remains a promising, yet hypothetical approach that requires validation.

## 4. Discussion

Researchers in clinical neurosciences are paying increasing attention to fluid biomarkers in neurodegenerative diseases, and they may offer valuable applications in HSP. In this narrative review we have recapitulated actual and potential fluid biomarkers in monitoring HSP disease, attempting a possible integrative framework to be tested in research studies despite the genetic and clinical heterogeneity of the clinical condition. Considerations from the aforementioned literature set out the rationale for selecting biomarkers in HSP, based on mechanistic and pharmacokinetic principles, and future directions centered on minimally invasive, patient-friendly monitoring are explored.

### 4.1. Biomarker Selection Strategy in HSP: Mechanistic and Pharmacokinetic Considerations

**Figure 1:**
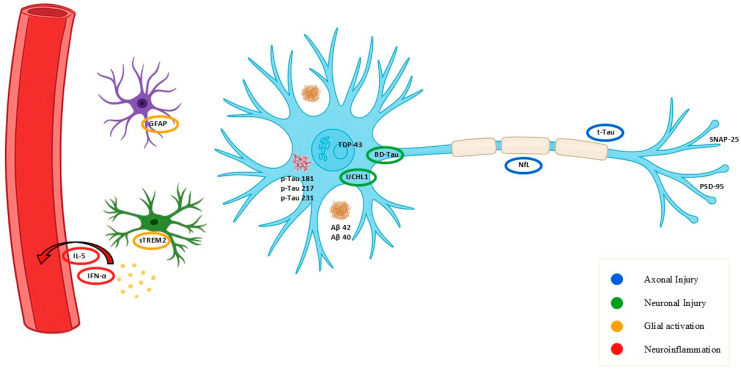
Schematic overview of key commercially available fluid biomarkers relevant to neurodegeneration and their cellular or molecular origin. Schematic representation of main fluid biomarkers and their source: NfL (axonal injury), BD-tau and UCHL1 (neuronal injury), GFAP and sTREM2 (glial activation), cytokines (IL-5, IFN-α; neuroinflammation), and synaptic proteins (SNAP-25, PSD-95). Alzheimer’s disease–related markers (pTau isoforms, Aβ40/42) are shown for comparison. Colors indicate biological process: blue = axonal injury, green = neuronal injury, orange = glial activation, red = neuroinflammation.

Mutations in over 90 HSP genes affect diverse cellular processes, including axonal transport, mitochondrial dynamics, and lipid metabolism. This heterogeneity makes mutation-specific biomarker development impractical, as it is difficult to develop a single biomarker that can be used for all gene variants. Shared downstream effects such as axonal degeneration, neuronal loss and glial activation should be the focus of biomarker strategies instead. These effects represent convergent and biologically meaningful targets across HSP subtypes (Figure 1).

In this review, we propose a multidimensional and integrative framework. Multidimensional implies the combinatory effects of three axes: biological domain (axonal, neuronal, glial, immune, synaptic), pharmacokinetic profile (e.g., half-life, persistence in circulation), and clinical context of use (diagnosis, progression, trial endpoints). Integrative indicates that, despite genetic heterogeneity, biomarkers can be mapped onto shared downstream pathways of neurodegeneration—axonopathy, neuronal loss, and glial activation. This framework is put forward as a practical strategy for biomarker selection and interpretation, rather than a unification of upstream disease-related mechanisms.

Commercially available fluid biomarkers relevant to HSP can be grouped into three mechanistic categories: axonal injury (e.g., NfL, tau), neuronal cell body damage (e.g., BD-tau, UCHL1), and glial/inflammatory activity (e.g., GFAP, sTREM2, cytokines). Among available biomarkers, NfL stands out as the most established in HSP. It reliably reflects axonal degeneration, matches the core pathophysiology of HSP, and offers suitable pharmacokinetics for tracking chronic progression. BD-tau, with high CNS specificity and minimal peripheral interference, complements NfL by capturing cortical and subcortical neuronal damage, particularly in complex HSP subtypes. Additionally, GFAP, sTREM2, and selected cytokines can detect short-lived inflammatory or glial activation events that may signal transient disease activity. These markers may uncover biologically relevant fluctuations not evident on routine clinical evaluation, providing a more dynamic view of HSP progression.

A bona fide multidimensional biomarker panel should include:

- NfL, to monitor chronic axonal damage;
- BD-tau, to assess CNS-specific neuronal injury;
- GFAP, cytokines, or sTREM2, to capture transient glial or immune activation.

**Figure 2:**
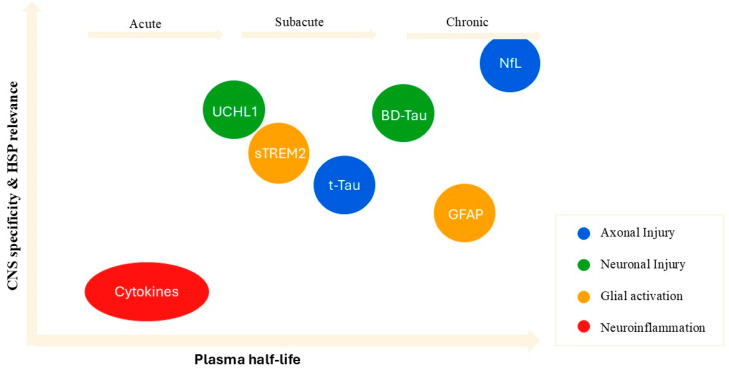
Spatiotemporal landscape of fluid biomarkers based on CNS specificity, HSP pathophysiological relevance, and plasma half-life. Biomarkers are positioned according to plasma half-life (x-axis) and CNS specificity/relevance to HSP (y-axis). Colors indicate biological processes: blue = axonal injury, green = neuronal injury, orange = glial activation, red = neuroinflammation.

This multi-dimensional biomarker strategy, informed by both the mechanistic relevance and the pharmacokinetic properties of each molecule (Figure 2), offers a rational and clinically feasible framework for monitoring disease activity in both symptomatic patients and asymptomatic carriers, guiding therapeutic decision-making across the continuum of HSP care.

### 4.2. Clinical Translation and Presymptomatic Carriers

The clinical translation of fluid biomarkers in HSP requires a careful and structured approach. For NfL, elevations are consistently observed in patients compared to controls, with levels influenced by age and sex and showing a rise around the time of phenotypic conversion before stabilizing thereafter. At present, no HSP-specific cut-offs are available, and interpretation should therefore rely on age-adjusted reference ranges and be expressed in relative terms such as z-scores or percentiles. Single measurements are less informative than longitudinal trajectories, particularly in presymptomatic carriers where only a sustained upward trend is likely to indicate disease activity. False positives may arise from comorbidities, systemic inflammation, trauma, or intense physical activity, underscoring the need for repeated sampling and contextual interpretation [87,88].

A particularly promising field of application is the monitoring of presymptomatic carriers identified through genetic testing [89]. While genetic diagnostics unequivocally identifies individuals at risk, it neither provides information on the biological activity of the disease nor predicts the timing of symptom onset. Fluid biomarkers may fill this gap by offering dynamic indicators of underlying neurodegenerative processes. For instance, serum NfL can reflect axonal injury and has already shown increases around the time of clinical conversion in gene carriers, whereas GFAP or sTREM2 may capture episodes of glial activation and neuroinflammation that precede or accompany onset. Embedding these biomarkers into longitudinal follow-up of carriers could therefore enable the detection of subclinical disease activity and provide early warning of phenoconversion. Beyond individual patient management, this combined genetic–biomarker approach may prove crucial for trial readiness, by identifying at-risk subjects in the earliest phases and allowing therapeutic interventions to be tested before irreversible neurodegeneration occurs.

### 4.3. Limitations

Despite the promise of fluid biomarkers in HSP, several methodological and conceptual limitations must be acknowledged. A primary challenge lies in the rarity and genetic heterogeneity of the disease. With over 90 causative genes identified, most available studies have concentrated on a narrow subset, typically SPG4 or SPG11, leaving the majority of genetic forms unexplored. As a consequence, findings cannot be readily generalized to the full spectrum of HSPs, and genotype-specific effects remain insufficiently characterized.

Sample sizes represent another major constraint. Cohorts are often small and heterogeneous, which limits statistical power and increases the risk of type I and type II errors. This issue is particularly evident when attempting to stratify analyses by clinical stage, genotype, or age at onset. The long, slowly progressive course of HSP further complicates interpretation, as biomarker levels may fluctuate depending on duration, severity, and rate of disease progression. Without sufficiently powered longitudinal datasets, it remains difficult to disentangle whether observed changes reflect true disease dynamics or variability inherent to the assays and cohorts studied. Demographic and clinical modifiers add further complexity. Age exerts a well-established influence on biomarkers such as NfL and GFAP, and sex differences have been documented in several studies [90,91,92]. Comorbidities, including vascular risk factors and other neurological conditions, may also contribute to biomarker variability. In blood-based assays, peripheral sources of proteins (e.g., systemic inflammation for GFAP, peripheral nerves for NfL) can obscure CNS-specific signals, underscoring the need for rigorous control groups and age-adjusted reference values.

Technical and analytical issues also remain a barrier to clinical translation. Inter-assay variability, pre-analytical handling, and limited assay standardization restrict comparability across studies [93,94]. Plasma-based measurements, while more accessible, generally show weaker dynamic ranges and specificity compared to CSF, raising questions about sensitivity in slowly progressive conditions such as HSP [95,96]. Moreover, normative datasets covering the pediatric to adult age span are still lacking, preventing robust determination of cut-offs or thresholds for clinical use.

Another important limitation is the interaction between biomarker dynamics and disease stage. HSPs are not uniform entities: they comprise multiple genetic and clinical subtypes, each potentially characterized by a different balance of axonal degeneration, neuronal loss, and glial or inflammatory activation. These components are unlikely to occur simultaneously with equal weight throughout the course of disease. For example, certain HSP forms may display a prominent glial or inflammatory component in early stages, while in advanced phases NfL levels may plateau as the axonal pool progressively declines and fewer fibers remain to release measurable protein. This temporal variability complicates interpretation and suggests that the translational meaning of a given biomarker is phase dependent. Addressing this challenge will require large-size, longitudinal studies that follow patients over years, ideally integrating fluid biomarkers with imaging modalities and clinical and digital assessments to obtain a multidimensional view of disease progression.

In addition to genetic mechanisms, microenvironmental factors may also influence pathogenic cascades in HSP. Changes in ionic strength, protein concentration, and divalent cations such as calcium or zinc can promote or stabilize protein aggregation [97,98]. Although not directly investigated in HSP, these processes could modulate disease progression and should be considered when interpreting biomarker dynamics.

Beyond these methodological aspects, regulatory translation represents an additional challenge. The qualification of biomarkers as drug development tools requires adherence to structured frameworks such as the FDA Biomarker Qualification Program (https://www.fda.gov/) and the EMA “Qualification of Novel Methodologies” initiative (https://www.ema.europa.eu/en/homepage). These programs demand a clearly defined context of use, rigorous analytical validation, and reproducibility across cohorts. In HSP, integration into such pathways would allow fluid biomarkers like NfL, BD-tau, or GFAP to progress beyond exploratory use and achieve formal recognition as enrichment factors, pharmacodynamic readouts, or even surrogate endpoints. This regulatory recognition is particularly relevant in the context of HSP trials, which face the obstacles of rarity, slow progression, and phenotypic heterogeneity. Qualified biomarkers could improve trial feasibility by enhancing patient stratification, reducing sample size requirements, and providing early indicators of treatment effect.

All in all, these limitations further emphasize the need for larger, multicenter longitudinal studies with harmonized protocols and standardized analytical workflows, coupled with systematic efforts to embed biomarker qualification within trial design. Only under these conditions will it be possible to establish reliable benchmarks and assess the true potential of fluid biomarkers in HSP.

### 4.4. Future Perspectives: Toward Patient-Centered, Remote Monitoring in HSP

Technological advances and a growing emphasis on patient quality of life are driving a paradigm shift in the management of chronic neurodegenerative conditions such as HSP. Care strategies are increasingly personalized and designed to be less physically burdensome for patients. In this context, the traditional model of requiring patients to visit specialized clinics for venous blood draws, often combined with clinical evaluations, may become obsolete.

**Figure 3:**
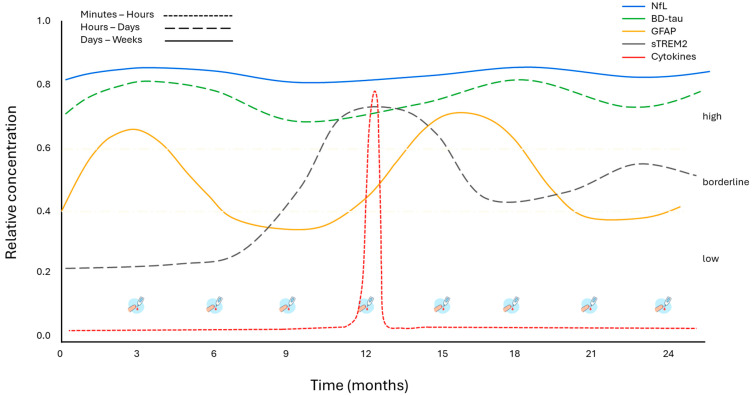
Hypothetical plasma biomarker dynamics over 24 months in HSP using dried blood spot (DBS) sampling. Conceptual model of plasma biomarkers over 24 months in HSP. The colors of the lines correspond to the individual biomarkers, while line style (continuous or dashed) indicates their half-life and persistence in circulation. References for biomarker half-life and persistence: [58,100,101,102,103,104,105,106,107,108]. DBS = dried blood spot.

Recent innovations have introduced dried blood spot (DBS) sampling kits, allowing for remote blood collection with minimal effort [99]. DBS involves collecting a single drop of blood from a fingertip prick, a procedure familiar to diabetic patients and easily performed without clinical supervision. This method enables frequent, home-based sampling and opens the door to more granular and cost-effective disease monitoring. The utility of DBS lies not only in improving patient comfort and accessibility but also in its ability to provide a richer temporal profile of biomarker fluctuations. Frequent, low-burden sampling may help capture transient changes in disease activity that would otherwise be missed with infrequent clinical assessments. This is particularly valuable in the context of biomarkers with short half-lives—such as inflammatory or glial markers—which can serve as sensitive indicators of subclinical or episodic changes in neurodegeneration within the broader chronic progression of HSP (Figure 3).

To translate recent biomarker advances into clinical practice, the development of harmonized, multicenter protocols is essential to ensure global data comparability and scientific rigor. A prime example of such standardization efforts is the SP-CERN (Spastic Paraplegia—Centers of Excellence Research Network) in the United States. This collaborative initiative includes over 11 clinical centers and collects standardized clinical, molecular, and digital data across sites, with centralized biobanking and harmonized workflows. SP-CERN provides an infrastructure for biomarker validation, natural history modeling, and future therapeutic trials in HSP. In Europe, networks such as TreatHSP, the Ataxia Global Initiative, and SPATAX are advancing similar goals by promoting shared clinical scales (e.g., SPRS), longitudinal biosample collection, and consensus-based protocols for genetic and phenotypic characterization. These initiatives aim to bridge methodological gaps and align biomarker research efforts across centers and countries. Integrating such harmonized multicenter frameworks with remote sampling technologies could facilitate a new generation of biomarker-guided care in HSP. This combined strategy offers scalability, geographic reach, and improved temporal resolution, enabling earlier and more equitable access to emerging therapeutic interventions. In parallel, coordination with FDA and EMA qualification programs will be essential to ensure that validated biomarkers can be adopted as regulatory-grade tools in future HSP trials.

## 5. Conclusions

This review provides a structured framework for considering fluid biomarkers in HSP, organized according to their underlying pathophysiological mechanism and pharmacokinetic properties, including half-life. The aim is to align biomarker selection with different phases of disease, distinguishing markers that capture long-term neurodegeneration from those that reflect short-lived inflammatory or glial activity. A central implication of this framework is that single measurements are rarely sufficient: repeated sampling is required to detect subtle and often subclinical changes that anticipate overt progression.

From a clinical translation standpoint, NfL remains the most established marker, but its interpretation depends on age- and sex-adjusted reference ranges and is most informative when assessed longitudinally. BD-tau adds CNS specificity and may complement NfL in tracking neuronal injury, while glial and immune markers have potential value in selected subtypes or time windows. Overall, the practical use of biomarkers in HSP should rest on three principles: standardized and adjusted reporting, integration with clinical/digital and imaging data, and prioritization of longitudinal over cross-sectional assessment to reduce misclassification, especially in presymptomatic carriers. By linking biomarker choice to disease mechanism, sampling frequency, and half-life, this framework may support a more phase-specific and biologically grounded approach to monitoring HSP. Through longitudinal and harmonized application, fluid biomarkers can begin to bridge the gap between genetic diagnosis and dynamic disease activity, providing a realistic basis for future personalized strategies in HSP.

## Figures and Tables

**Table 2:** Biomarkers in Neurodegeneration: Localization, Function, and Clinical Utility in HSP Context.

Mechanistic Category	Biomarker	Localization	Use in HSP	Strength of Evidence in HSP	Key References
**Amyloid**	Aβ40/Aβ42	CNS, peripheral tissues	No utility; amyloid not involved	Preliminary in HSP	[23,24]
**Tau** (neuronal soma/axonal)	t-tau, p-tau (181/217/231), BD-tau	Neuron soma and axons	Limited role in HSP; BD-tau may hold potential in complex/advanced subtypes	Preliminary in HSP (t-Tau and p-Tau); Speculative (BD-Tau)	[23,25,26,27,28]
**Axonal injury**	Neurofilament light chain (NfL)	Large-caliber myelinated axons	Elevation in SPG4; correlates with progression	Established in HSP	[29,30,31,32]
**Glial/inflammatory**	GFAP, sTREM2	Astrocytes (GFAP)/Microglia (sTREM2)	Not relevant in pure HSP; possible in inflammatory/complex forms	Preliminary in HSP (GFAP); Speculative (sTREM2)	[32,33,34,35,36,37]
**Neuronal integrity/stress**	UCH-L1, TDP-43	Neurons and glia	Exploratory in HSP; potential in complex/overlapping phenotypes	Speculative	[38,39,40,41]
**Synaptic**	SNAP-25, PSD-95	Pre- and postsynaptic compartments	Not studied in HSP; theoretical interest	Speculative	[42,43,44]

Abbreviations: Aβ: Amyloid Beta; CNS: Central Nervous System BD-tau: Brain-Derived Tau; NfL: Neurofilament Light Chain; GFAP: Glial Fibrillary Acidic Protein; sTREM2: Soluble Triggering Receptor Expressed on Myeloid Cells 2; UCH-L1: Ubiquitin Carboxy-terminal Hydrolase L1; TDP-43: TAR DNA-Binding Protein 43; SNAP-25: Synaptosomal-Associated Protein 25; PSD-95: Postsynaptic Density Protein 95. Classification reflects the level of evidence in HSP: established = case–control studies with concordant results; preliminary = single or discordant studies; speculative = mechanistic considerations without supporting studies.

## Data Availability

No datasets were generated or analyzed during the preparation of this review.
